# Supplementary material for: Pathophysiological Significance of Neutrophilic Transfer RNA-Derived Small RNAs in Asymptomatic Moyamoya Disease
Source: Cells. 2021 May 1;10(5):1086. doi: 10.3390/cells10051086 (PMC8147334; doi:10.3390/cells10051086)

2D Structure	Local AU	Position

```
31                          8mer-1a       56
5'-agGTGTCCTCG-GGCT-CA-TATGTGGGa-3'  utr
     |  :||||  :  |||    |  :|||||||
3'-ucCUUAGGAUUGGCGAUCUGGUACACCCu-5'  tRF
               16151413   7 6 5 4 3 2
             3' Pairing      Seed
```

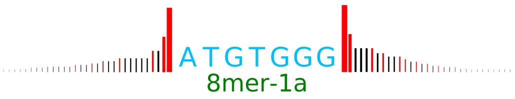

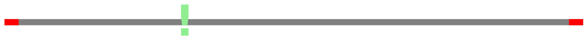

Supplement: Supplementary file 1 [file cells-10-01086-s001.zip › cells-1149161 3rd supplementary materials/Data Supplements/Data Supplement V.zip!/Data supplement 6-mRNAs targets of tsRNAs/tsRNA-Glu-TTC-010/Diagrams/tRF-Glu-TTC-010_vs_ENST00000327097(MARCO).pdf]
